# High‐production dairy cattle exhibit different rumen and fecal bacterial community and rumen metabolite profile than low‐production cattle

**DOI:** 10.1002/mbo3.673

**Published:** 2018-09-11

**Authors:** Yingyu Mu, Xueyan Lin, Zhonghua Wang, Qiuling Hou, Yun Wang, Zhiyong Hu

**Affiliations:** ^1^ College of Animal Science Shandong Agricultural University Taian Shandong China

**Keywords:** bacterial diversity, high‐production dairy cows, low‐production dairy cows, metabolites, rumen fluid

## Abstract

Our aim was to simultaneously investigate the gut bacteria typical characteristic and conduct rumen metabolites profiling of high production dairy cows when compared to low‐production dairy cows. The bacterial differences in rumen fluid and feces were identified by 16S rDNA gene sequencing. The metabolite differences were identified by metabolomics profiling with liquid chromatography mass spectrometry (LC‐MS). The results indicated that the high‐production dairy cows presented a lower rumen bacterial richness and species evenness when compared to low‐production dairy cows. At the phylum level, the high‐production cows increased the abundance of Proteobacteria and decreased the abundance of Bacteroidetes, SR1, Verrucomicrobia, Euryarchaeota, Planctomycetes, Synergistetes, and Chloroflexi significantly (*p* < 0.05). At the genus level, the rumen fluid of the high‐production group was significantly enriched for *Butyrivibrio*,* Lachnospira*, and *Dialister* (*p* < 0.05). Meanwhile, rumen fluid of high‐production group was depleted for *Prevotella, Succiniclasticum, Ruminococcu, Coprococcus,* YRC22, CF231, 02d06, *Anaeroplasma, Selenomonas*, and *Ruminobacter* significantly (*p* < 0.05). A total of 92 discriminant metabolites were identified between high‐production cows and low‐production cows. Compared to rumen fluid of low‐production dairy cows, 10 differential metabolites were found up‐regulated in rumen fluid of high‐production dairy cows, including 6alpha‐Fluoropregn‐4‐ene‐3,20‐dione, 3‐Octaprenyl‐4‐hydroxybenzoate, disopyramide, compound III(S), 1,2‐Dimyristyl‐sn‐glycerol, 7,10,13,16‐Docosatetraenoic acid, ferrous lactate, 6‐Deoxyerythronolide B, vitamin D2, L‐Olivosyl‐oleandolide. The remaining differential metabolites were found down‐regulated obviously in high‐production cows. Metabolic pathway analyses indicated that most increased abundances of rumen fluid metabolites of high‐yield cows were related to metabolic pathways involving biosynthesis of unsaturated fatty acids, steroid biosynthesis, ubiquinone and other terpenoid‐quinone biosynthesis. Most down‐regulated metabolic pathways were relevant to nucleotide metabolism, energy metabolism, lipid metabolism and biosynthesis of some antibiotics.

## 1 INTRODUCTION

Many studies showed that the gut microbiota play an important role on health, metabolism and immunity of the host recently (Amato et al., 2014; Ridaura et al., 2013; Trompette et al., 2014). The bovine rumen harbors a symbiotic microbiota capable of converting indigestible plant mass into energy (Flint, Bayer, Rincon, Lamed, & White, 2008), which is of vital importance for production of milk and meat. Among this complex microbial community, 95% of the microorganisms are bacteria (Brulc et al., 2009). Jami, White, and Mizrahi (2014); recently identified the connections between the ratio of Firmicutes to Bacteroides and daily milk‐fat yield. Variation in the rumen microbiome of dairy cattle has also been correlated with methane emission levels (Ross, Moate, Marett, Cocks, & Hayes, 2013b), and metagenomic profiling of the rumen microbiome can actually be used to predict phenotypes relating to enteric methane gas production (Ross, Moate, Marett, Cocks, & Hayes, 2013a). These studies revealed connections between the gut microbial community and certain physiological host parameters, which can be applied on improving animal production by manipulation of relevant beneficial bacterial flora.

Some studies suggested correlations between rumen microbial groups and bovine feed efficiency traits (Carberry, Kenny, Han, McCabe, & Waters, 2012; Guan, Nkrumah, Basarab, & Moore, 2008; Hernandez‐Sanabria et al., 2012). The relative proportion of sequences that were assigned to *Prevotella* appeared to be positively associated with high residual feed intake (RFI) in bulls, whereas an unidentified group within the order Bacteroidales was positively associated with low RFI in bulls (McCann et al., 2014). Lima et al. (2015) have characterized the rumen fluid microbiomes of prepartum and postpartum high‐producing Holstein cows and revealed that some bacteria have strong correlations with milk production. Moreover, they built a multivariable regression model using bacterial taxa significantly associated with average milk yield in the first 150 days postpartum to predict the weekly milk production; this microbiome‐predicted milk yield was significantly correlated with the actual weekly averages of milk production.

Additionally, an early study on mice observed that obese mice (ob/ob) exhibited a different ratio of the phyla Firmicutes to Bacteroidetes when compared with lean littermates (Ley et al., 2005). This difference is not totally attributable to differences in food consumption, as a runted ob/ob mouse weighed less than the ob/ob littermates owing to reduced chow consumption, but still demonstrated a significantly greater percent body fat and ratio of Firmicutes to Bacteroidetes. Moreover, analogous differences have been observed in the distal gut microbiota of obese versus lean humans as well; the relative abundance of Bacteroidetes increases as obese individuals lose weight on either a fat‐ or a carbohydrate‐restricted low calorie diet. And the increase in Bacteroidetes was markedly relevant to weight loss but not to total caloric intake. (Ley, Turnbaugh, Klein, & Gordon, 2006). A later study by the same team demonstrated that the obesity‐associated gut microbiome have increased capacity for energy harvest by transplantation of lean and obese cecal microbiotas into germ‐free wild‐type mouse recipients (Turnbaugh et al., 2006)

However, there is still a lack of information investigating the gut bacterial profile of high‐production dairy cows. In this work, we explored the bacterial differences in rumen fluid and feces from high‐yield and low‐yield dairy cows under the same diet, region and surroundings. Meanwhile, we investigated the metabolites differences in rumen fluid between these two groups as well. The aim of the study was to explore the rumen and fecal bacteria, and the rumen metabolite profiles of high‐production dairy cows. Bacteria which are associated with high‐production dairy cattle have the potential to be cultured and applied as probiotics to improve the performance of low‐production dairy cattle.

## 2 MATERIALS AND METHODS

### 2.1 Experimental animals and sample collection

We selected 22 Holstein dairy cows, with 11 high‐yield and 11 low‐yield cows; 11 of each were matched as pairs, and each pair of cows was reared under the same diet regimes, feeding environment, and were paired for similar age, parity and nearing lactation days (Table 1) on the aote dairy farm in Qingdao. Cows were fed twice (05:00 hr and 17:00 hr) and milked twice daily; all cows had free access to clean water.

**Table 1:** Fundamental information of cows in experiment

High‐yield cows				Low‐yield cows			
Cow	Milk yield (kg)	Parity	Lactation days	Cow	Milk yield (kg)	Parity	Lactation days
9,052	38	3	182	10,067	18	3	168
10,019	38	2	216	11,063	21	2	224
10,091	46	3	119	10,045	27	3	91
11,030	48	2	93	11,091	28	2	72
11,089	31	1	489	11,093	18	1	505
12,006	37	1	413	12,002	24	1	441
12,017	42	1	405	12,010	27	1	387
12,031	40	1	223	12,040	25	1	257
12,073	40	1	186	12,068	25	1	191
12,083	41	1	214	12,092	28	1	201
13,021	35	1	64	13,042	20.7	1	54

Representative rumen fluid samples were obtained from all Holstein cows via the cow's mouth with the oro‐ruminal sampling device within two hours before morning feeding. Fecal samples were acquired by the rectum pick dung method. All samples were immediately placed into liquid nitrogen, and were transferred to laboratory for −80°C storage.

The ethics committee of Shandong agriculture university approved the study (SDAU2015‐18).

### 2.2 Experimental procedures for 16S rDNA sequencing

The samples were slowly thawed at 4°C. Total DNA was extracted from the rumen fluid and fecal samples using the Stool DNA Isolation Kit (Tiangen, Beijing, China). DNA samples were quantified, using a Nanodrop spectrophotometer (Nyxor Biotech, Paris, France), and then transferred to BGI Genomics for V4 region of the 16S rDNA gene sequencing with PE250 Miseq. The PCR primer used for 16S rDNA amplicon libraries was 515F‐806R.

### 2.3 Bioinformatics analysis for 16S rDNA sequencing

The raw data were filtered to eliminate the adapter pollution and low quality to obtain clean reads (Douglas et al., 2014). Truncation of sequence reads not having an average quality of 20 over a 30 bp sliding window based on the Phred algorithm, and trimmed reads having less than 75% of their original length, as well as its paired read, were removed. Then paired‐end reads with overlap were merged into tags by FLASH (Magoc & Salzberg, 2011) (Fast Length Adjustment of Short reads, v1.2.11). Tags were clustered to OTU at 97% sequence similarity by scripts of software USEARCH (v7.0.1090) (Edgar, 2013). Chimeras were filtered out by using UCHIME (v4.2.40) (Edgar, Haas, Clemente, Quince, & Knight, 2011). OTU representative sequences were taxonomically classified using the Ribosomal Database Project (RDP) Classifier v.2.2 (Cole et al., 2014) trained on the Greengenes database (V201305) (DeSantis et al., 2006) using 0.5 confidence values as cutoff. OTUs were filtered to remove unassigned OTUs.

VennDiagram and package ‘ade4’ of software R (v3.0.3) were used separately in Venn diagram and OTU PCA analysis. The tags number of each taxonomic rank (phylum, class, order, family, genus and species) or OTU in different samples were summarized in a profiling table. The species with abundances less than 0.5% were classified into ‘others’ in other ranks for all samples.

The representative sequences were aligned against the Silva core set(Silva_108_core_aligned_seqs), using PyNAST by ‘align_seqs.py’. The indices of Alpha diversity were calculated by Mothur (v1.31.2), and the corresponding rarefaction curves were drawn by R (v3.0.3). Wilcoxon Rank‐Sum Test was used for comparison of two groups, using the alpha diversity indices. Beta diversity analysis was done by software QIIME(v1.80) (Caporaso et al., 2010). PCoA (Principal coordinate analysis) is used to exhibit the differences between the samples according to the matrix of beta diversity distance. Unweighted Pair Group Method with Arithmetic mean (UPGMA) is a type of hierarchical clustering method using average linkage and was used to interpreting the distance matrix produced by beta diversity. To measure the robustness of this result to sequencing effort, we perform a jackknifing analysis, wherein 75% of the smallest sample sequences from each sample are chosen at random, and the resulting UPGMA tree from this subset of data is compared with the tree representing the entire available data set by QIIME(v1.80). This process is repeated with 100 random subsets of data, and the tree nodes which prove more consistent across jackknifed datasets are deemed more robust. And the figure is drawn by software R(v3.0.3).

The abundance differences in microbial communities between samples were obtained using statistical methods, and FDR (false discovery rate) was adopted to assess the significance of differences. Metastats (http://metastats.cbcb.umd.edu/) and R (v3.0.3) were used to determine which taxonomic groups were significantly different between groups of samples. We adjusted the obtained *p*‐value by a Benjamini–‐Hochberg false discovery rate correction (function ‘p.adjust’ in the stats package of R (v3.0.3)) (James, Niranjan, & Mihai, 2009).

### 2.4 Metabolomics profiling with liquid chromatography mass spectrometry (LC‐MS)

#### 2.4.1 Metabolites extraction

Metabolites extraction is achieved by organic solvent to precipitate protein (Sarafian et al., 2014). A quantity of 100 μl liquid sample with micropipette was plunged into 96‐well plate. 300 μl isopropanol (−20°C) was added to the well above and vortex mixed and then stored overnight at −20°C. Centrifuged at 14,000*g* for 20 min at 4°C and collected supernatants to new tube until LC‐MS analysis.

QC: Pooling of sample supernatants (5 μl from every sample).

#### 2.4.2 Bioinformatics analysis for LC‐MS

For metabolomics profiling, we used Xevo G2‐XS QTOF (Waters, U.K.) to detect metabolites in the samples. Progenesis QI software (Waters, U.K. http://www.nonlinear.com/progenesis/qi/) was used for the preprocessing and identification steps. Each detected metabolic feature was normalized to the QC sample using LOESS Signal Correction (LSC). The correction effect is evaluated by RSD analysis and PCA analysis. The stability of the analytical system is evaluated by intensity distribution of QC samples. Ions with no signal(intensity equals 0) in more than 80% samples of any group are discarded. The above ions with RSD <30% are included for further analysis. All filtered metabolomics data were searched against the KEGG database. For statistics analysis, we used multivariate analysis (PCA/PLS‐DA) and univariate analysis (FDR/Fold change) to gain differential ions. Cluster analysis was generated using the ‘pheatmap’ package in R (v3.0.3). Pathway analysis was performed through the KEGG database.

## 3 RESULTS

### 3.1 Sequencing quality

A total of 3,214,190 paired reads were retained with an average of 73,049 reads per sample. A total of 3,183,371 tags were obtained, with 72,349 tags per sample on average and an average length of 252 bp.

The rarefaction curve based on Observed species value, Chao1 value and ACE values suggest all samples produced sufficient data (Supporting Information, Figure S1).

### 3.2 OTU classification and statistics

Based on the OTU abundances, OTUs of each group were listed. The number of OTUs found in HighS, LowS, HighF, and LowF were 2,329, 2,592, 2,265, and 2,574, respectively. A total of 1,929 OTUs were shared between HighS and LowS; similarly, 1851 OTUs were shared between HighF and LowF (Supporting Information Figure S2).

**Figure 1:**
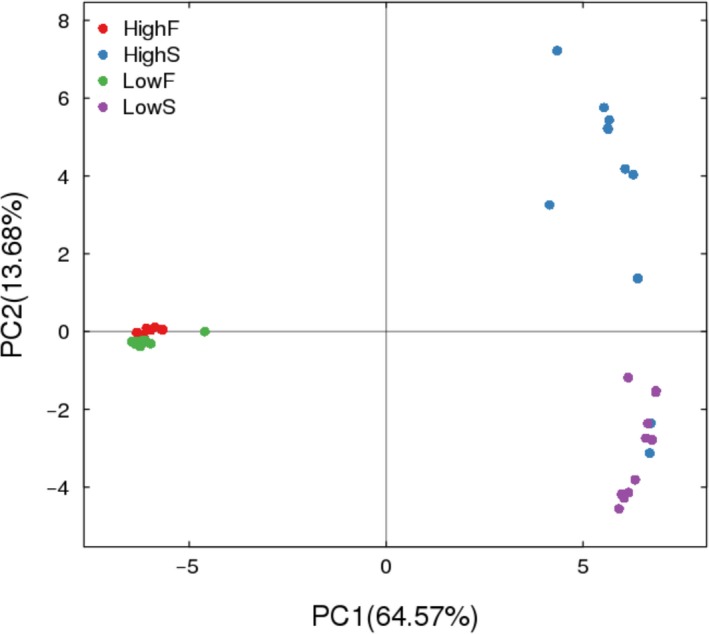
PCA analyses of bacteria in rumen fluid and fecal samples. ***Note:*** Unweighted UniFrac was used to create the PCA. *X*‐axis, 1st principal component; *Y*‐axis, 2nd principal component. The number in brackets represents the contributions of principal components to differences among samples. A dot represents each sample, and different colors represent different groups. HighS and LowS represent rumen fluid from high‐yield and low‐yield cows, respectively. HighF and LowF represent groups of high‐yield and low‐yield cow feces, respectively

The OTU composition was distinctly different between HighS and LowS, while there was no significant difference between HighF and LowF (Figure 1).

**Figure 2:**
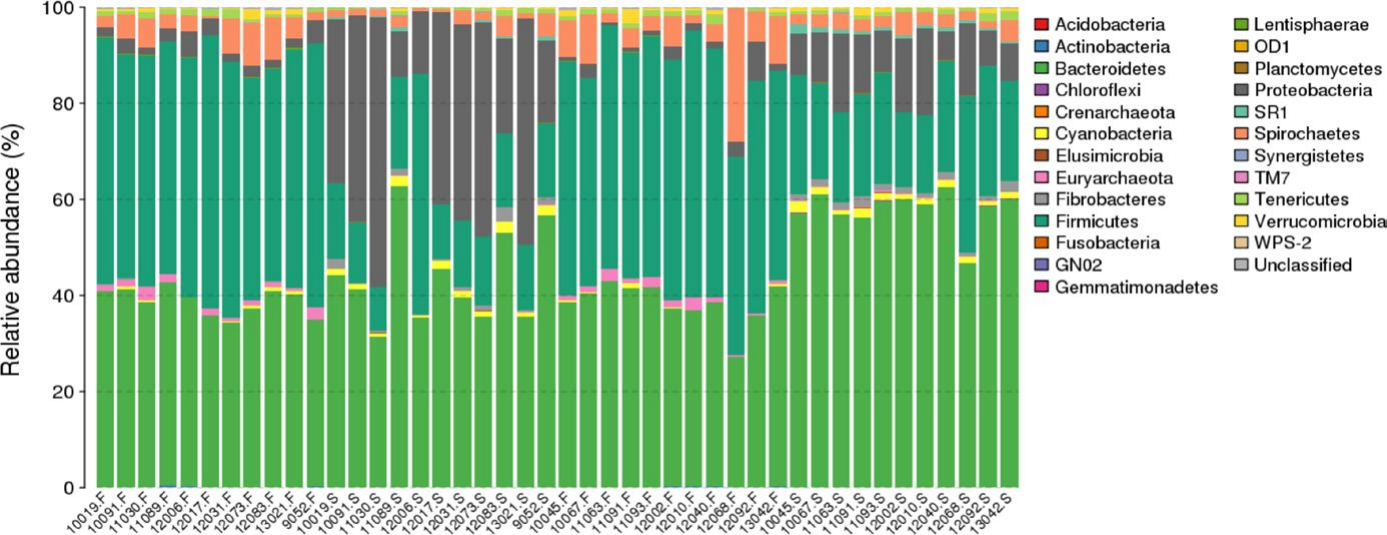
The taxonomic composition distribution in rumen fluid and fecal samples at the phylum level. ***Note.*** Abscissa represent samples of rumen fluid or feces and each serial number represent a different cow. S represents rumen fluid samples. F represents fecal samples. The vertical axis represent relative abundance of each phylum

For the rumen fluid samples, a total of 20 phyla were identified, and the predominant phyla the species of whose abundance was more than 1% were Bacteroidetes, Proteobacteria, Firmicutes, Spirochaetes, and Cyanobacteria (Figure 2). As for the fecal samples, a total of 22 phyla were identified, and the predominant phyla were Bacteroidetes, Firmicutes, Spirochaetes, Proteobacteria, Euryarchaeota and Tenericutes (Figure 2).

At the genus level, a total of 100 and 96 genera were detected in the HighS and LowS groups, respectively. Genera with abundances greater than 1% in the HighS group included *Prevotella, Lachnobacterium, Succiniclasticum, Treponema, Ruminococcus*, and *Butyrivibrio*. For the LowS group, the corresponding genera consisted of *Prevotella, Succiniclasticum, Ruminococcus, Treponema, YRC22, Fibrobacter, CF231*,* and Coprococcus* (Supporting Information Figure S3).

A total of 95 and 114 genera were detected in the HighF and LowF groups, respectively. Genera with abundances greater than 1% in the HighF and the LowF were almost identical, including 5‐7N15, CF231, *Treponema, Oscillospira, Prevotella, Coprococcus, Methanobrevibacter*, and *Paludibacter*. There were four more genera in the HighF compared to LowF and they were *Phascolarctobacterium, Anaerostipes Ruminobacter*, and *Ruminococcus* (Supporting Information Figure S3).

### 3.3 Diversity analysis within and among samples

Alpha diversity (Patrick et al., 2009) was applied for analyzing the complexity of species diversity of a sample through several indices, including the Chao1, ACE, Shannon and Simpson indices. The sample complexity was proportional with the first four values, and negatively correlated with the Simpson value. The observed species, Chao1 and ACE values can reflect the species community richness, and the rarefaction curve based on the three values can also be used to evaluate whether the produced data was sufficient to cover all species within the community. The Shannon and Simpson values reflected the species diversity of the community, affected by both species richness and species evenness, that was the two values also consider the abundance of each species.

There were significant differences in both species richness and species evenness between HighS and LowS. LowS samples had a higher richness and species evenness than HighS samples (Table 2). But there were no significant differences in richness or evenness between HighF and LowF (Table 3).

**Table 2:** Alpha diversity indices of rumen fluid between high‐production dairy cows and low‐production dairy cows

Alpha	Mean (HighS)	*SD* (HighS)	Mean (LowS)	*SD* (LowS)	*p*‐vaule
sobs	870.56	281.37	1,326.44	109.46	0.00049
chao	1,019.7	302.19	1,442.51	83.04	0.00049
Ace	1,028.4	306.66	1,456.25	85.62	0.00049
shannon	3.36	0.78	5.21	0.28	0.00016
simpson	0.19	0.08	0.02	0.01	0.00016

***Note.*** HighS represents for rumen fluid of high‐production cows. LowS represents for rumen fluid of low‐production cows. Wilcoxon Rank‐Sum Test is used for two group comparation. If *p* value is less than 0.05, there is significant difference in alpha diversity between the two groups.

**Table 3:** Alpha diversity indices of fecal samples between high‐production dairy cows and low‐production dairy cows

Alpha	Mean (HighF)	*SD* (HighF)	Mean (LowF)	*SD* (LowF)	*p*‐vaule
sobs	1,056.67	143.78	1,209.78	195.99	0.09391
chao	1,170.56	150.69	1,291.75	186.44	0.16153
ace	1,182.41	156.08	1,309.81	188.94	0.11349
shannon	5.16	0.25	5.25	0.28	0.34011
simpson	0.02	0.01	0.02	0.01	0.48943

***Note.*** HighF represents for fecal samples of high‐production cows. LowF represents for fecal samples of low‐production cows. Wilcoxon Rank‐Sum Test is used for a two group comparison. If *p* value is less than 0.05, there is significant difference in alpha diversity between the two groups.

**Figure 3:**
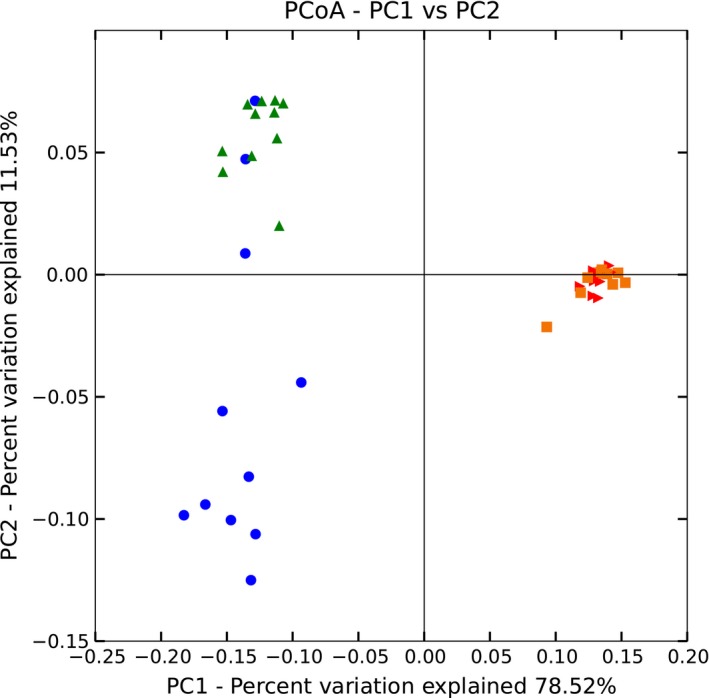
Weighted unifrac PCoA analyses of bacteria in rumen fluid and fecal samples. ***Note.*** Green triangles represent rumen fluid of high‐sproduction cows. Blue dots represent rumen fluid of low‐production cows. Red triangles represent fecal samples of high‐production cows. Orange squares represent fecal samples of low‐production cows

**Figure 4:**
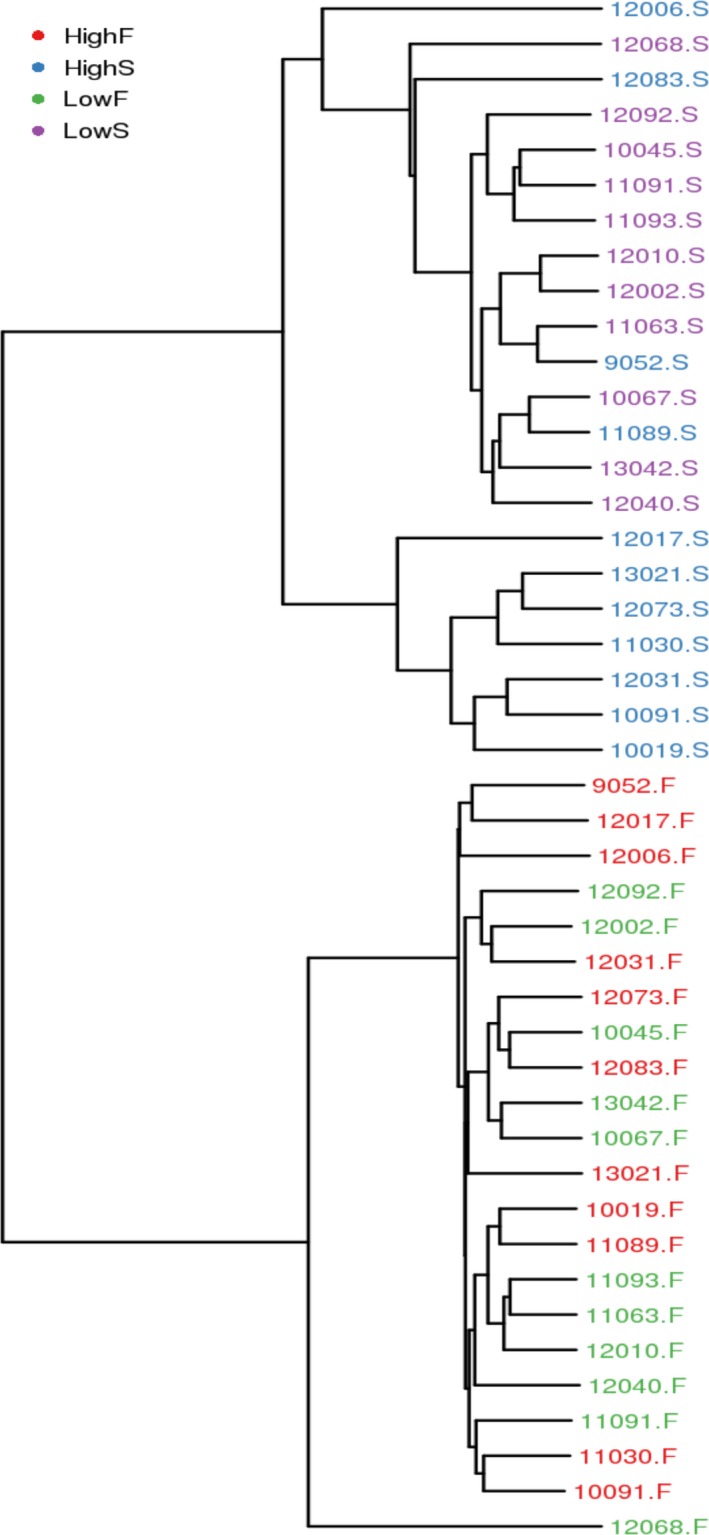
Weighted_unifrac cluster tree of rumen fluid and fecal samples. ***Note.*** The same color represents the samples in the same group. Short distance between samples represents high similarity. HighS and LowS represent rumen fluid from high‐yield and low‐yield cows, respectively. HighF and LowF represent groups of high‐yield and low‐yield cow feces, respectively

Beta diversity analysis was used to evaluate sample differences in species complexity. PCoA (Principal coordinate analysis) was used to exhibit the differences between the samples according to the matrix of beta diversity distances. PCoA analysis and the clustering results showed that bacterial communities in the rumen fluid were separated from those in the feces (Figures 3 and 4). There were marked differences between HighS and LowS but almost no differences between HighF and LowF samples (Figures 3 and 4).

### 3.4 Significant differences analysis between groups of samples

Significant bacterial differences were identified at the level of phylum, class, order, family, genus and species between HighS and LowS, HighF and LowF.

At the phylum level, 0.04% and 0.10% of the respective phyla were unclassified in the HighS and LowS groups, and these differences were not significant (*p *>* *0.05). Compared to low‐yield rumen fluid group, the high‐yield group was significantly enriched for the phylum Proteobacteria (*p *<* *0.05). Abundances were significantly lower for phyla Bacteroidetes, SR1, Verrucomicrobia, Euryarchaeota, Planctomycetes, Synergistetes, and Chloroflexi (*p *<* *0.05) (Table 4). Among the fecal samples, the respective unclassified ratios for HighF and LowF were 0.32% and 0.30%. There were no significant differences in the relative abundances of each phylum between HighF and LowF (*p *>* *0.05) either.

**Table 4:** The significant bacterial differences in rumen fluid at the level of phylum

Phylum	Mean (HighS)	Std.err (HighS)	Mean (LowS)	Std.err (LowS)	*p*‐vlaue	FDR
Bacteroidetes	43.72	3.01	58.00	1.25	0.003	0.010
Proteobacteria	33.21	4.73	11.40	1.27	0.001	0.010
SR1	0.26	0.09	0.76	0.11	0.002	0.010
Verrucomicrobia	0.18	0.07	0.60	0.14	0.012	0.031
Euryarchaeota	0.10	0.02	0.22	0.03	0.008	0.024
Planctomycetes	0.04	0.01	0.11	0.01	0.003	0.010
Synergistetes	0.02	0.01	0.05	0.00	0.002	0.010
Chloroflexi	0.01	0.00	0.04	0.01	0.002	0.010

***Note.*** HighS represents for rumen fluid of high‐production cows. LowS represents for rumen fluid of low‐production cows. Metastats is used for a two group comparison study. If *p* value is less than 0.05, there is significant difference in alpha diversity between the two groups.

**Figure 5:**
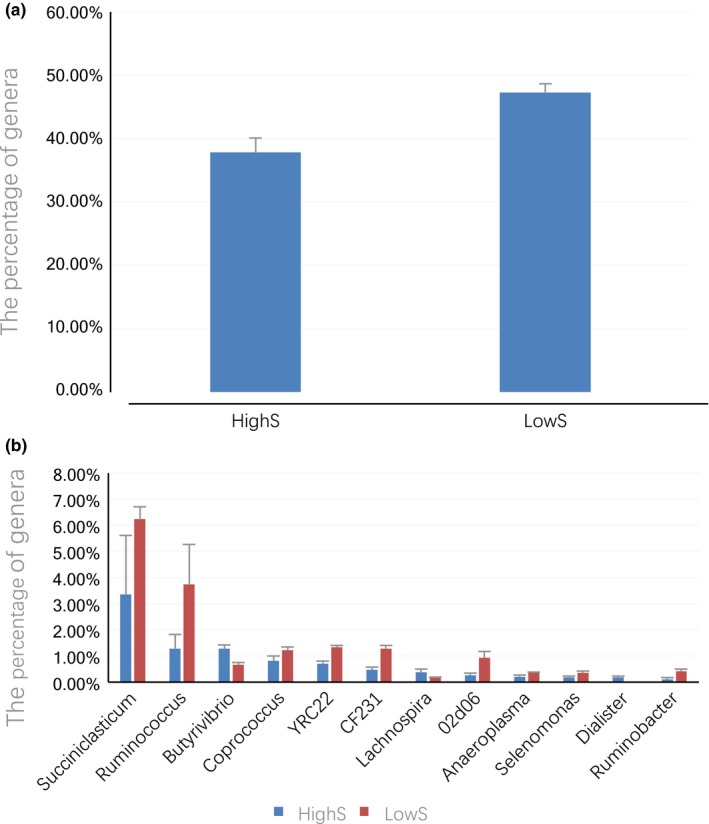
(a) The difference of Prevotella in rumen fluid between high‐production cows and low‐production cows. (b) The taxonomic distribution of genera differing significantly in abundance among rumen fluid samples. ***Note.*** HighS represents for rumen fluid of high‐production cows. LowS represents for rumen fluid of low‐production cows

At the genus level, the relative abundances of unclassified genera in HighS and LowS were 44.8% and 30.7% (*p *>* *0.05), respectively. Then we analyzed the genera whose proportion was greater than or equal to 0.1% in the rumen fluid and feces. Compared to LowS, the high‐yield group had significantly fewer *Prevotella* in Bacteroidetes, *Succiniclasticum* in Firmicutes, *Ruminococcus* in Firmicutes, *Coprococcus* in Firmicutes, *YRC22* in Bacteroidetes, *CF231* in Bacteroidetes, *02d06* in Firmicutes, *Anaeroplasma* in Tenericutes, *Selenomonas* in Firmicutes and *Ruminobacter* in Proteobacteria (*p *<* *0.05). The HighS group was significantly enriched for *Butyrivibrio* in Firmicutes, *Lachnospira* in Firmicutes and *Dialister* in Firmicutes (*p *<* *0.05, Figure 5a,b).

**Figure 6:**
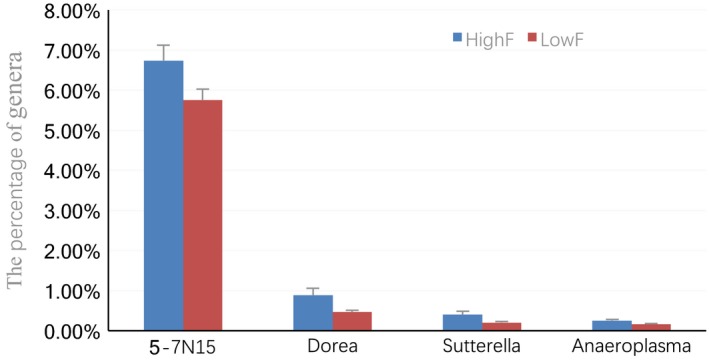
Distribution of taxonomic compositions for significantly different genera in fecal samples. *Note*. HighF represents for fecal samples of high‐production cows. LowF represents for fecal samples of low‐production cows

There were no significant differences in enrichment for unclassified genera (*p *>* *0.05) between the HighF and LowF groups, (64.68% and 64.51%, respectively). Compared to LowF, the high‐yield group was significantly enriched for genera *5‐7N15*,* Dorea*,* Sutterella*, and *Anaeroplasma* (*p *<* *0.05) among the classified genera (Figure 6).

### 3.5 Metabolic profiling

**Figure 7:**
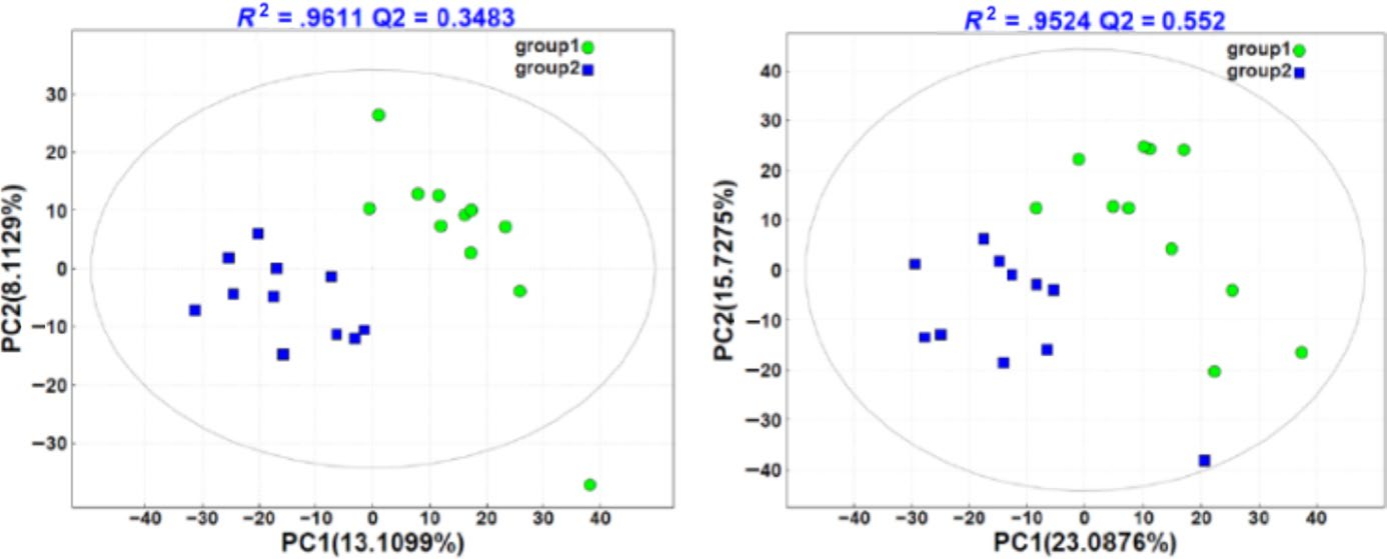
PLS‐DA score plot of rumen metabolites between groups. *Note*. The abscissa represents the first principal component PC1, the ordinate represents the second principal component PC2, and the model parameter R2 is above the graph. Each point in the plot corresponds to an observation. The groups are shown in different colors. Group 1 represents high‐production cows. Group 2 represents low‐production cows

The PLS‐DA model showed a clear separation of samples between high‐yield and low‐yield dairy cows (Figure 7). The most discriminant metabolites were selected by filtering for fold changes of >1.2 or <0.8, simultaneous with q‐value of <0.05 and vip of >1.0.

**Figure 8:**
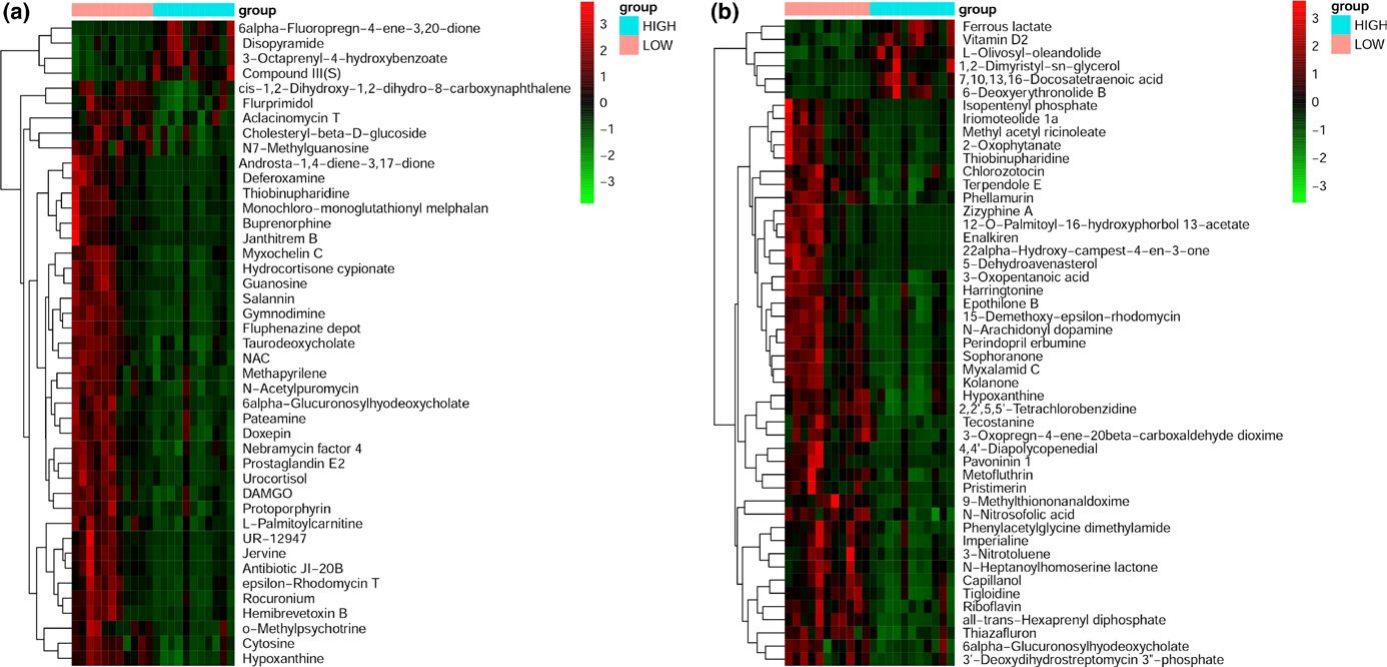
(a) Heat map analysis of significant differences in abundance of metabolites in negative ion mode. (b) Heat map analysis of significant differences in abundance of metabolites in positive ion mode

A total of 92 discriminant metabolites were identified between high‐production and low‐production cows. Compared with low‐production dairy cows, totally 10 differential metabolites were found to be up‐regulated in high‐production dairy cows, including 6alpha‐Fluoropregn‐4‐ene‐3,20‐dione, 3‐Octaprenyl‐4‐hydroxybenzoate, disopyramide, compound III(S), 1,2‐Dimyristyl‐sn‐glycerol, 7,10,13,16‐Docosatetraenoic acid, ferrous lactate, 6‐Deoxyerythronolide B, vitamin D2, and L‐Olivosyl‐oleandolide (Figure 8a,b). And most abundant changes were related to metabolic pathways, involving biosynthesis of unsaturated fatty acids, steroids, ubiquinone and other terpenoid‐quinones, and biosynthesis of 12‐, 14‐, and 16‐membered macrolides (Table 5). A total of 82 different metabolites were found to be down‐regulated in high‐yield cows compared to low‐yield cows. Detailed information of these metabolites and their corresponding metabolic pathways are presented in Table 6.

**Table 5:** Discriminant metabolites with up‐regulated in the rumen fluid of high‐production dairy cows and their corresponding metabolic pathways

Metabolic pathway	Metabolite	VIP	fold_change	*q* value
Biosynthesis of 12‐, 14‐ and 16‐membered macrolides	L‐Olivosyl‐oleandolide;	1.804	0.692	0.031
	6‐Deoxyerythronolide B;	2.783	0.397	0.019
Biosynthesis of unsaturated fatty acids	7,10,13,16‐Docosatetraenoic acid	2.966	0.374	0.032
Steroid biosynthesis	Vitamin D2	2.095	0.582	0.036
Ubiquinone and other terpenoid‐quinone biosynthesis	3‐Octaprenyl‐4‐hydroxybenzoate	1.532	0.543	0.034
Biosynthesis of antibiotics	L‐Olivosyl‐oleandolide;	1.804	0.692	0.031
	6‐Deoxyerythronolide B;	2.783	0.397	0.019

**Table 6:** Discriminant metabolites with down‐regulated in the rumen fluid of high production dairy cows and their corresponding metabolic pathways

Metabolic pathway	Metabolite	VIP	fold_change	*q* value
Purine metabolism	Hypoxanthine	2.845	2.343	0.012
	Guanosine;	2.867	2.974	0.018
Riboflavin metabolism	Riboflavin	1.884	1.482	0.021
Indole diterpene alkaloid biosynthesis	Terpendole E;	1.922	1.498	0.031
Glucosinolate biosynthesis	9‐Methylthiononanaldoxime;	2.687	2.983	0.019
Brassinosteroid biosynthesis	22alpha‐Hydroxy‐campest‐4‐en‐3‐one	3.840	14.100	0.017
Terpenoid backbone biosynthesis	Isopentenyl phosphate;	3.386	6.262	0.017
	All‐trans‐Hexaprenyl diphosphate;	2.653	2.184	0.014
Neuroactive ligand‐receptor interaction	N‐Arachidonyl dopamine	2.831	2.515	0.017
Biosynthesis of type II polyketide products	15‐Demethoxy‐epsilon‐rhodomycin;	2.682	2.429	0.044
	Epsilon‐Rhodomycin T;	2.658	3.818	0.025
	Aclacinomycin T	2.073	1.633	0.046
Carotenoid biosynthesis	4,4’‐Diapolycopenedial;	2.980	3.655	0.017
Biosynthesis of alkaloids derived from terpenoid and polyketide	Jervine;	3.338	6.084	0.012
	Thiobinupharidine;	2.890	3.084	0.016
Pyrimidine metabolism	Cytosine;	3.018	3.160	0.019
Arachidonic acid metabolism	Prostaglandin E2	3.037	4.148	0.025
Porphyrin and chlorophyll metabolism	Protoporphyrin	2.098	1.879	0.048
Fatty acid degradation	L‐Palmitoylcarnitine;	3.103	4.497	0.007
Steroid degradation	Androsta‐1,4‐diene‐3,17‐dione;	4.446	5.758	0.013
Puromycin biosynthesis	N‐Acetylpuromycin;	2.512	2.196	0.016
Steroid hormone biosynthesis	Urocortisol	3.370	4.597	0.016
Naphthalene degradation	cis‐1,2‐Dihydroxy‐1,2‐dihydro‐8‐carboxynaphthalene;	2.155	1.631	0.036
Biosynthesis of antibiotics	Antibiotic JI‐20B	3.551	9.685	0.029
	Epsilon‐Rhodomycin T;	2.658	3.818	0.025
	N‐Acetylpuromycin;	2.512	2.196	0.016
	Nebramycin factor 4;	2.598	2.060	0.031
	Aclacinomycin T	2.073	1.633	0.046
Steroid biosynthesis	5‐Dehydroavenasterol;	3.654	11.265	0.023
Ubiquinone and other terpenoid‐quinone biosynthesis	All‐trans‐Hexaprenyl diphosphate;	2.653	2.184	0.014

## 4 DISCUSSION

In agreement with other previous studies, the three dominant phyla observed in all rumen fluid samples were Bacteroidetes, Firmicutes, and Proteobacteria. Contrasted with the higher ratio of Firmicutes to Bacteroidetes in feces, the abundance of Firmicutes in rumen fluid was far less than that of Bacteroidetes, which was consistent with other studies (Jami, Israel, Kotser, & Mizrahi, 2013). It was known that the fiber content in the rumen was far higher than that in the hindgut; thus, we inferred the extra Bacteroidetes present in rumen fluid may be enriched for cellulolytic bacteria. Analogous differences were observed in a recent study on goat (Do et al., 2018). The study indicated that increasing the members of Bacteroidetes to keep low ratio of Firmicutes versus Bacteroidetes in goat rumen resulted an increased lignocellulose digestion. More interestingly, the high‐production cows showed a significant increase in phylum Proteobacteria compared to low‐production cows. In fact, the abundance of Proteobacteria in the high‐production group was even greater than that of Firmicutes, demonstrating a reversed result with the low‐production group and with most previous studies (Jami et al., 2014; Jewell, McCormick, Odt, Weimer, & Suen, 2015). However, when we further analyzed at the genus level, any genus within Proteobacteria that could account for the marked differences between the two groups was detected, which could be explained by the high unclassified ratio (31%–45%). Much more work still needs to be done to investigate the genus‐level difference in phylum Proteobacteria between the high and low‐production dairy cows.

Among the identified genera, *Prevotella* represented the highest percentage in spite of the milk production. The abundance of *Prevotella* in the high‐yield group (37.85%) was lower than that in the low‐yield group significantly (47.29%). *Prevotella* was found negatively associated with RFI in dairy cows (Jami et al., 2014), and the same study also suggested there was a strong negative correlation (Pearson R = −0.69, *p* =  5 × 10^−3^) between *Prevotella* and milk‐fat yield. Moreover, a study on Korean Adolescents showed that *Prevotella* was associated with triglycerides (TG) and total cholesterol positively, and ultimately induced obesity (Hu et al., 2015). In our study, we did not measure the milk fat ratio of the cows. But the low production cows were fatter than the high production cows generally.

The HighS group was significantly enriched for the genera *Butyrivibrio*,* Lachnospira* and *Dialiste*r when compared with low‐yield group. The genera *Butyrivibrio* and *Lachnospira* both belong to the Family Lachnospiraceae. In the rumen, some special strains of *Butyrivibrio fibrisolvens* degrade cellulose completely and quickly. *Lachnospira sp*. are mostly involved with pectin degradation (Cotta & Forster, 2006). Lima et al. (2015) revealed a positive correlation with *Butyrivibrio* abundance and milk yield. Jami et al. (2014) showed a positive correlation between *Dialister* and milk yield. *Ruminococcus, Coprococcus* and *Succiniclasticum* were suggested to have negative connections with milk production (Jami et al., 2014), which was consistent with our results. Very few studies referred to the other significant bacteria such as YRC22, CF231 and 02d06, all with completely unknown and unexplored functions in rumen physiology.

Compared to low‐production dairy cows, pathway analyses indicated that most abundant up‐regulation changes in high‐yield cows were related to metabolic pathways involving biosynthesis of unsaturated fatty acids, steroid biosynthesis, ubiquinone and other terpenoid‐quinone biosynthesis. Boerman and Lock (2014) suggested unsaturated fatty acids (UFA) treatments supplemented at 2% of diet DM as either soybean FA distillate or soybean oil increased milk yield, but did not effectively reduce milk fat yield. Ubiquinone had been suggested to play an important role in the mitochondrial generation of hydrogen peroxide (Boveris, Cadenas, & Stoppani, 1976). However, the metabolic pathways of reduced abundance metabolites in high‐production dairy cows were mainly relevant to nucleotide metabolism, energy metabolism, lipid metabolism and biosynthesis of some antibiotics.

The microbiome interacted with the host immune system to regulate metabolism by various mechanisms: direct physical contact, production of metabolites, and shedding of structural components (Zmora at al., 2017). These affected metabolic homeostasis by local mucosal immune modulation and by remote alteration of metabolic organs, such as adipose tissue, muscle, and the liver. It was a pity that we did not detect immune indicators in this study. So we are planning to explore the differences in the blood immunity indices between high‐production and low‐production dairy cows in the following study.

## 5 CONCLUSION

In this study, significant bacterial differences were presented between high‐yield and low‐yield dairy cows, which were mainly reflected by the relative abundances of some special bacteria. Furthermore, there existed significant metabolic differences including biosynthesis of unsaturated fatty acids, steroid biosynthesis, energy metabolism, fatty acid metabolism, amino acid metabolism, biosynthesis of some antibiotics, etc. between the two groups. However, much more work still needs to be done to identify the detailed differences in bacterial abundances between high‐yield and low‐yield dairy cows. Accordingly, we can isolate specific beneficial dominant strains in high production cows sequentially to provide material for carrying out microorganism mediated nutritional regulation.

## CONFLICT OF INTEREST

The authors declare no conflict of interest.
