# Supplementary figures and images for: High‐production dairy cattle exhibit different rumen and fecal bacterial community and rumen metabolite profile than low‐production cattle
Source: Microbiologyopen. 2018 Sep 11;8(4):e00673. doi: 10.1002/mbo3.673 (PMC6460281; doi:10.1002/mbo3.673)

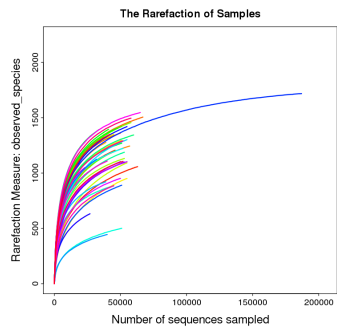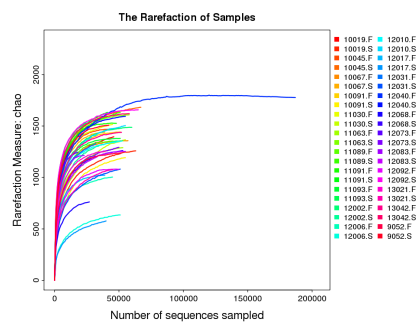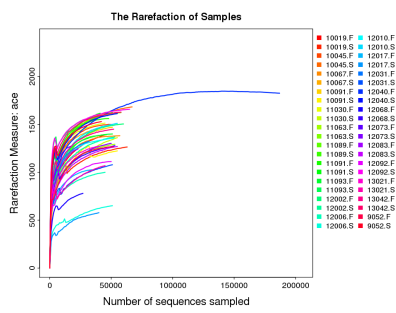

Supplement: Supplementary file 1 [file MBO3-8-e00673-s001.pdf]

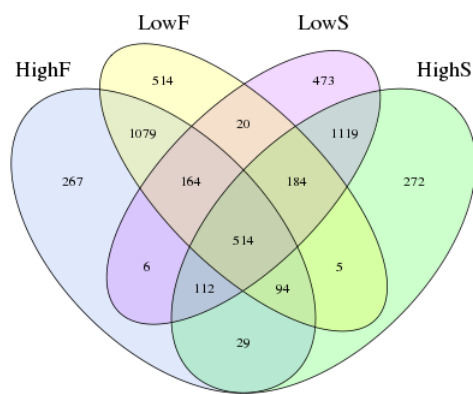

Supplement: Supplementary file 2 [file MBO3-8-e00673-s002.pdf]

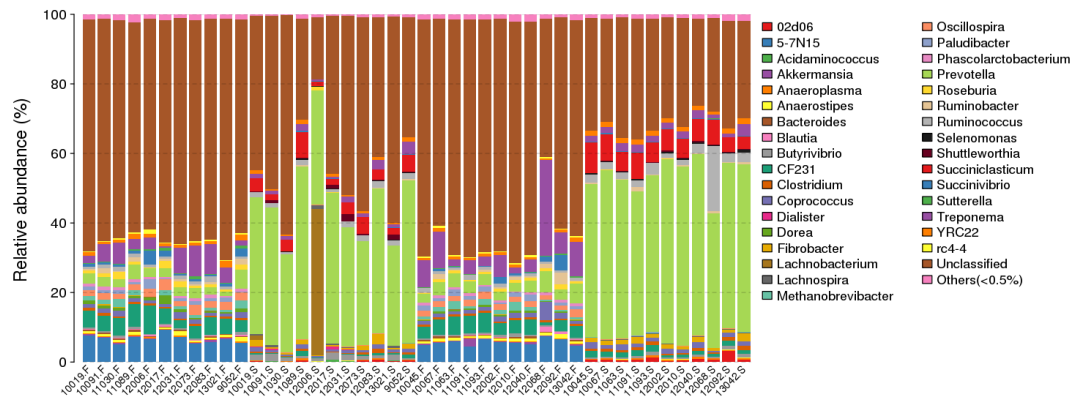

Supplement: Supplementary file 3 [file MBO3-8-e00673-s003.pdf]
